# Prognostic impact of regional lymph node metastasis at castration-resistant prostate cancer diagnosis on survival after radium-223 treatment

**DOI:** 10.1093/jjco/hyag125

**Published:** 2026-08-07

**Authors:** Saizo Fujimoto, Takuma Kato, Masatoshi Eto, Mototaka Sato, Takahito Negishi, Akinori Wada, Tadamasa Shibuya, Jun Akatsuka, Shuichi Morizane, Tatsuya Takayama, Akira Tsujimura, Hiroki Kitou, Hiromasa Tsukino, Yoshihiro Matsunaga, Kazutoshi Fujita, for the FRONTIER Study Group, Akinori Wada, Akira Tsujimura, Atsushi Kyan, Atsushi Mizokami, Dai Koguchi, Fumio Ishizaki, Fumio Tsukuda, Hirofumi Izaki, Hirofumi Morinaka, Hiroki Kitou, Hiromasa Tsukino, Hiroshi Kitamura, Hisanori Taniguchi, Jun Akatsuka, Junichi Inokuchi, Junya Furukawa, Katsuyoshi Hashine, Kazutoshi Fujita, Kohei Hasimoto, Koichiro Wada, Koji Shiraishi, Kazuki Kokura, Eiji Matsubara, Masashi Kato, Masashi Morita, Masatoshi Eto, Masaya Ito, Masayoshi Nagata, Masayuki Hagiwara, Minato Yokoyama, Mitsuru Noguchi, Naoki Fujita, Naoki Terada, Nobuhiro Haga, Norihiko Kawamura, Norihiko Tsuchiya, Norio Nonomura, Mototaka Sato, Osamu Ukimura, Rei Unno, Ryoichi Imamura, Ryoma Kurahashi, Ryuji Matsumoto, Ryuto Nakazawa, Sadanori Kamikawa, Sayuri Takahashi, Seiji Hoshi, Shingo Sugaya, Shinichi Sakamoto, Shinsuke Kurokawa, Hiromi Sato, Shuichi Tatarano, Soichi Makino, Shuichi Morizane, Tadamasa Shibuya, Takahito Negishi, Takanori Mochizuki, Takashi Fukagai, Takashi Kawahara, Takashi Kobayashi, Takashi Saika, Tatsuya Takayama, Toru Harabayashi, Toshiaki Shinojima, Toshitaka Shin, Atsuro Sawada, Tsutomu Nishiyama, Yasuo Kohjimoto, Yasushi Nakai, Yoshihiro Hasegawa, Yoshihiro Matsunaga, Yoshiyuki Miyazawa, Yujiro Nagata, Yuki Yamada, Yukio Naya, Yusuke Ito, Yutaka Yamamoto

**Affiliations:** Department of Urology, Kindai University Faculty of Medicine, Osaka, Japan; Department of Urology, Kagawa University Faculty of Medicine, Kagawa, Japan; Department of Urology, Kyushu University Graduate School of Medical Sciences, Fukuoka, Japan; Department of Urology, Toyonaka Municipal Hospital, Osaka, Japan; Department of Urology, National Hospital Organization Kyushu Cancer Center, Fukuoka, Japan; Department of Urology, Shiga University of Medical Science, Shiga, Japan; Department of Urology, Japanese Redcross Oita Hospital, Oita, Japan; Department of Urology, Nippon Medical School, Tokyo, Japan; Division of Urology, Department of Surgery, Faculty of Medicine, Tottori University, Tottori, Japan; Department of Urology, International University of Health and Welfare Nasu Medical Center, Nasushiobara, Japan; Department of Urology, Juntendo University Urayasu Hospital, Chiba, Japan; Department of Urology, Japan Community Health Care Organization Tokyo Shinjuku Medical Center, Tokyo, Japan; Department of Urology, Junwakai Memorial Hospital, Miyazaki, Japan; Department of Urology, Kurume University School of Medicine, Fukuoka, Japan; Department of Urology, Kindai University Faculty of Medicine, Osaka, Japan

**Keywords:** radium-223, castration-resistant prostate cancer, regional lymph node metastasis, overall survival, real-world evidence

## Abstract

**Background:**

Radium-223 (Ra-223) is used for patients with bone-metastatic castration-resistant prostate cancer (CRPC). However, the prognostic value of regional lymph node metastasis at CRPC diagnosis for survival after Ra-223 remains unclear. This study evaluated the association between regional lymph node metastasis at CRPC diagnosis and overall survival (OS) after Ra-223 initiation.

**Methods:**

This multicenter retrospective cohort study used the Following Ra-223 in Outcomes after New-generation Therapy with Investigation of Effects in Refractory prostate cancer study database. Patients with bone-metastatic CRPC who initiated Ra-223 between January 2020 and December 2023 were included. Patients were classified according to the presence or absence of regional lymph node metastasis at CRPC diagnosis. The primary endpoint was OS from Ra-223 initiation. Cox proportional hazards regression was used to evaluate the association between regional lymph node metastasis and OS.

**Results:**

Among 802 patients who initiated Ra-223, 718 were included: 505 without regional lymph node metastasis and 213 with regional lymph node metastasis at CRPC diagnosis. Median OS was 31 months (95% confidence interval [CI], 26–35) and 17 months (95% CI, 16–22), respectively (log-rank *P* < 0.001). In multivariable analysis, regional lymph node metastasis at CRPC diagnosis was associated with poorer OS (hazard ratio, 1.396; 95% CI, 1.092–1.785; *P* = 0.008).

**Conclusions:**

Regional lymph node metastasis at CRPC diagnosis was associated with poorer OS after Ra-223 initiation. This status may provide prognostic information when considering subsequent treatment sequencing in bone-metastatic CRPC.

## Teaser

Regional lymph node metastasis at castration-resistant prostate cancer diagnosis was associated with poorer survival after radium-223 initiation.

## Introduction

Radium-223 (Ra-223) is a bone-targeted alpha-emitting therapy for patients with metastatic castration-resistant prostate cancer (mCRPC) and bone metastases. In the Alpharadin in Symptomatic Prostate Cancer Patients (ALSYMPCA) trial, Ra-223 improved overall survival (OS) and delayed symptomatic skeletal events in patients with mCRPC, symptomatic bone metastases, and no known visceral metastases [1, 2]. Ra-223 has since been used as a treatment option for selected patients with bone-metastatic mCRPC. More recently, combination strategies involving Ra-223 and androgen receptor pathway inhibition have also been evaluated in patients with bone-metastatic mCRPC [3]. These developments underscore the need for clinical information that can support prognostic assessment and treatment planning before Ra-223 selection.

Clinical outcomes after Ra-223 vary across patients. Previous studies have reported that performance status, bone disease burden, alkaline phosphatase (ALP), hemoglobin, lactate dehydrogenase, prostate-specific antigen (PSA), pain, treatment line, and completion of Ra-223 therapy are associated with survival after Ra-223 treatment [4–7]. Some real-world studies have also evaluated lymphadenopathy or lymph node metastasis as a prognostic factor near the time of Ra-223 initiation or study registration [8, 9]. These reports support the importance of disease status near Ra-223 initiation. However, they do not fully address whether regional lymph node metastasis at the time of CRPC diagnosis provides prognostic information for later outcomes after Ra-223.

Regional lymph node metastasis at CRPC diagnosis may reflect disease biology at the emergence of castration resistance. This time point precedes later Ra-223 selection and may help identify patients with a more aggressive disease course before subsequent treatment decisions are made. Regional lymph node metastasis should be distinguished from distant lymph node metastasis, which corresponds to M1a disease and represents systemic metastatic spread. Clarifying the prognostic significance of regional lymph node metastasis at CRPC diagnosis may therefore help clinicians interpret nodal status within the broader treatment sequence for mCRPC.

This study evaluated whether regional lymph node metastasis at CRPC diagnosis was associated with OS after Ra-223 initiation in patients with bone-metastatic CRPC using the Following Ra-223 in Outcomes after New-generation Therapy with Investigation of Effects in Refractory prostate cancer (FRONTIER) real-world database. The primary exposure was regional lymph node metastasis at CRPC diagnosis, corresponding to N1 disease. Distant lymph node metastasis was not included in the primary exposure definition and was evaluated separately as metastatic disease.

## Materials and methods

### Study design and patients

This multicenter retrospective cohort study was performed using the database of the FRONTIER study. The FRONTIER study was designed to evaluate outcomes after Ra-223 treatment in patients with CRPC and bone metastases who were treated in routine clinical practice in Japan.

The present study was a subanalysis of the overall Ra-223-treated cohort of the FRONTIER study. The source population consisted of patients with pathologically confirmed prostate cancer who had bone-metastatic CRPC and initiated Ra-223 between 1 January 2020 and 31 December 2023, at participating institutions. This analysis used the overall FRONTIER cohort to evaluate the prognostic impact of regional lymph node metastasis at CRPC diagnosis, rather than a subgroup defined by prior upfront therapy or concomitant treatment during Ra-223 therapy.

The present analysis included patients who received at least one cycle of Ra-223 and had available data on regional lymph node status at CRPC diagnosis and survival outcomes after Ra-223 initiation. Clinical information was collected retrospectively from medical records. The collected data included baseline clinicopathological features, previous treatments, disease status at CRPC diagnosis, disease status at Ra-223 initiation, laboratory findings, Ra-223 treatment status, treatment outcomes, adverse events, and survival outcomes.

Data were managed using an Electronic Data Capture system based on Research Electronic Data Capture. Each patient was assigned a study identification number, and directly identifiable personal information was not entered into the study database.

### Inclusion and exclusion criteria

Patients were eligible for this analysis if they met all of the following criteria: age 18 years or older, pathologically confirmed prostate cancer, diagnosis of CRPC, bone-metastatic CRPC treated with Ra-223 between 1 January 2020 and 31 December 2023, receipt of at least one cycle of Ra-223, available regional lymph node status at CRPC diagnosis, available date of Ra-223 initiation, and available OS time and OS event status.

Patients were excluded if regional lymph node status at CRPC diagnosis, the date of Ra-223 initiation, OS time, or OS event status was unavailable. Patients with insufficient date records for survival analysis were also excluded.

### Exposure variable

The primary exposure variable was regional lymph node metastasis at CRPC diagnosis. Regional lymph node metastasis corresponded to N1 disease at CRPC diagnosis. Patients were classified into two groups according to the presence or absence of regional lymph node metastasis at CRPC diagnosis. Patients without regional lymph node metastasis were classified as the CRPC N0 group, and those with regional lymph node metastasis were classified as the CRPC N1 group.

Distant lymph node metastasis, corresponding to M1a disease, was not included in the primary exposure definition. Distant lymph node metastasis was summarized separately as metastatic disease and was considered in the interpretation of the results.

Regional lymph node metastasis at CRPC diagnosis was determined by local investigators based on imaging findings obtained in routine clinical practice. Imaging modalities that could be used for disease assessment included bone scintigraphy, computed tomography, magnetic resonance imaging, and positron emission tomography/computed tomography. The specific imaging modality used to determine regional lymph node status was not captured in the study database. Central radiologic review was not performed. The imaging modality and size criteria for regional lymph node metastasis were not centrally standardized.

### Endpoints

The primary endpoint was OS from Ra-223 initiation. OS was defined as the time from the first administration of Ra-223 to death from any cause or the date of last follow-up.

Secondary endpoints included cancer-specific survival (CSS), completion of six cycles of Ra-223, PSA response, ALP response, radiographic response, pain response, grade ≥ 3 haematologic toxicity, and symptomatic skeletal event (SSE). CSS was defined as the time from the first administration of Ra-223 to death from prostate cancer or the date of last follow-up. Completion of Ra-223 was defined as completion of six cycles. PSA response was defined as a decrease of ≥50% from the PSA level at Ra-223 initiation. ALP response was defined as an ALP decline of ≥30% after Ra-223 initiation. Objective response on imaging was defined as complete response or partial response, and disease control on imaging was defined as complete response, partial response, or stable disease. SSE was defined as external-beam radiotherapy for skeletal symptoms, new symptomatic pathological bone fracture, spinal cord compression, or orthopedic surgical intervention for bone metastasis.

Adverse events were graded according to the Common Terminology Criteria for Adverse Events version 5.0.

### Covariates

Covariates for the multivariable Cox proportional hazards model were selected based on clinical relevance and previously reported prognostic factors for OS after Ra-223 treatment. The multivariable model included regional lymph node metastasis at CRPC diagnosis, Gleason score sum, clinical T stage at diagnosis, performance status at Ra-223 initiation, extent of disease (EOD) score at Ra-223 initiation, PSA at Ra-223 initiation, ALP at Ra-223 initiation, hemoglobin at Ra-223 initiation, treatment line of Ra-223, and age at Ra-223 initiation.

Gleason score sum was modeled per 1-point increase. Clinical T stage was categorized as clinical T stage ≥3 versus <3 at diagnosis. Performance status was categorized as ≥1 versus 0. EOD score was categorized as ≥3 versus <3. PSA at Ra-223 initiation was log10-transformed; therefore, the hazard ratio represents the effect per 10-fold increase. ALP was modeled per 100 IU/L increase. Haemoglobin was modeled per 1 g/dl increase. Treatment line was categorized as Ra-223 administered as ≥3rd-line versus ≤2nd-line CRPC treatment. Age was modeled per 1-year increase.

Completion of Ra-223 was not included as a baseline covariate in the primary OS model because it was determined after treatment initiation.

### Statistical analysis

Patient characteristics were summarized according to regional lymph node status at CRPC diagnosis (Table 1). Continuous variables were presented as median and interquartile range. Categorical variables were presented as number and percentage. Between-group comparisons were performed using the Mann–Whitney U test for continuous variables and the chi-square test or Fisher’s exact test for categorical variables, as appropriate.

**Table 1:** Patient characteristics according to regional lymph node status at CRPC diagnosis.

	CRPC N0 (*n* = 505)	CRPC N1 (*n* = 213)	*P* value
At initial diagnosis			
Age, years	69.8 [64.7–75.5]	69.0 [64.0–75.0]	0.295
PSA, ng/ml	73.8 [14.2–405]	177 [36.8–814]	<0.001
Gleason score ≥ 9, n/N (%)	277/505 (54.9)	147/213 (69.0)	<0.001
cT3–4, n/N (%)	332/505 (65.7)	172/213 (80.8)	<0.001
M1, n/N (%)	305/501 (60.9)	178/212 (84.0)	<0.001
At CRPC diagnosis			
Progression from mHSPC to CRPC, n/N (%)	354/493 (71.8)	185/210 (88.1)	<0.001
Time from mHSPC diagnosis to CRPC, months	22.0 [11.0–52.0]	14.0 [8.00–23.0]	<0.001
cT3–4, n/N (%)	138/505 (27.3)	116/213 (54.5)	<0.001
M1, n/N (%)	415/503 (82.5)	195/213 (91.5)	0.002
Bone metastasis, n/N (%)	404/505 (80.0)	184/213 (86.4)	0.042
Visceral metastasis, n/N (%)	10/505 (2.0)	10/213 (4.7)	0.043
At Ra-223 initiation			
Time from CRPC diagnosis to Ra-223 initiation, months	16.0 [3.00–33.0]	17.0 [5.00–32.0]	0.85
Ra-223 administered as ≤2nd-line CRPC treatment, n/N (%)	273/504 (54.2)	99/213 (46.5)	0.06
Age, years	75.7 [70.9–81.3]	73.2 [68.1–78.6]	<0.001
Performance status ≥1, n/N (%)	250/505 (49.5)	117/213 (54.9)	0.184
PSA, ng/ml	8.66 [2.31–34.3]	17.1 [3.40–72.6]	0.002
ALP, IU/L	86.5 [62.0–131]	105 [74.5–175]	<0.001
Hemoglobin, g/dl	12.4 [11.3–13.3]	12.4 [11.3–13.3]	0.984
EOD score ≥ 3, n/N (%)	119/505 (23.6)	63/213 (29.6)	0.091
Pain at Ra-223 initiation, n/N (%)	139/486 (28.6)	72/206 (35.0)	0.097

Values are shown as median [interquartile range] or n/N (%), unless otherwise indicated. Patients were classified according to the presence or absence of regional lymph node metastasis at CRPC diagnosis. The CRPC N0 group included patients without regional lymph node metastasis at CRPC diagnosis, and the CRPC N1 group included patients with regional lymph node metastasis at CRPC diagnosis. Continuous variables were compared using the Mann–Whitney U test. Categorical variables were compared using the chi-square test or Fisher’s exact test, as appropriate. Distant lymph node metastasis, corresponding to M1a disease, was not included in the primary exposure definition.

Abbreviations: ALP, alkaline phosphatase; CRPC, castration-resistant prostate cancer; cT, clinical T stage; EOD, extent of disease; Hb, hemoglobin; IQR, interquartile range; M1, distant metastasis; mHSPC, metastatic hormone-sensitive prostate cancer; N1, regional lymph node metastasis; PSA, prostate-specific antigen; Ra-223, radium-223.

OS and CSS were estimated using the Kaplan–Meier method. Survival curves were compared between groups using the log-rank test. The association between regional lymph node metastasis at CRPC diagnosis and OS after Ra-223 initiation was evaluated using Cox proportional hazards regression models. Hazard ratios (HRs), 95% confidence intervals (CIs), and *P* values were calculated.

The primary multivariable analysis was performed using complete cases. The multivariable Cox model included 617 patients with available data for all covariates in the model. Patients with missing data for one or more model covariates were excluded from the multivariable analysis. Missingness by covariate was summarized in the Results section and in the footnote to Table 2. As a sensitivity analysis, the primary multivariable Cox model was additionally adjusted for distant lymph node metastasis at CRPC diagnosis, corresponding to M1a disease.

The proportional hazards assumption was assessed using Schoenfeld residuals. All statistical tests were two-sided, and *P* values <0.05 were considered statistically significant. Statistical analyses were performed using R version 4.4.1.

## Results

### Patient selection

Among 802 patients enrolled in the FRONTIER study who initiated Ra-223 between January 2020 and December 2023, 84 were excluded from the present analysis. The reasons for exclusion were missing regional lymph node status at the diagnosis of CRPC in 82 patients and insufficient date records in 2 patients. The final analytic cohort consisted of 718 patients, including 505 patients without regional lymph node metastasis at CRPC diagnosis (CRPC N0 group) and 213 patients with regional lymph node metastasis at CRPC diagnosis (CRPC N1 group) (Fig. 1).

**Figure 1:**
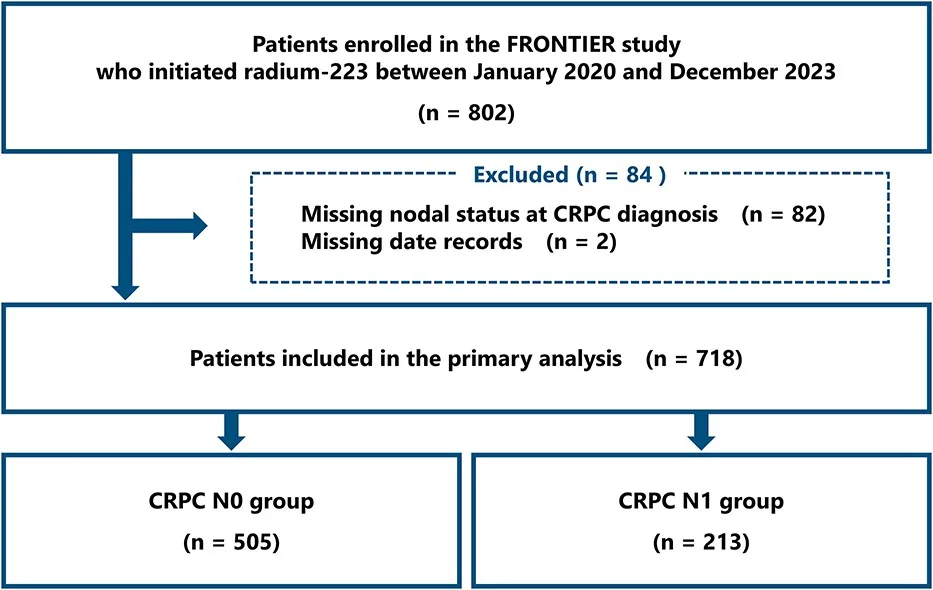
Patient flow diagram. Patient flow diagram for the present analysis. Among 802 patients enrolled in the FRONTIER study who initiated radium-223 (Ra-223) between January 2020 and December 2023, 84 patients were excluded because of missing regional lymph node status at the diagnosis of castration-resistant prostate cancer (CRPC) or insufficient date records. The final analytic cohort included 718 patients, comprising 505 patients without regional lymph node metastasis at CRPC diagnosis (CRPC N0 group) and 213 patients with regional lymph node metastasis at CRPC diagnosis (CRPC N1 group). Abbreviations: CRPC, castration-resistant prostate cancer; Ra-223, radium-223.

### Patient characteristics

Patient characteristics according to regional lymph node status at CRPC diagnosis are shown in Table 1. The CRPC N1 group had more advanced disease features than the CRPC N0 group at initial diagnosis, including higher PSA level, more frequent Gleason score ≥ 9, clinical T3–4 disease, and M1 disease.

At CRPC diagnosis, the CRPC N1 group had a shorter time from metastatic hormone-sensitive prostate cancer diagnosis to CRPC and higher frequencies of clinical T3–4 disease, M1 disease, bone metastasis, and visceral metastasis than the CRPC N0 group. Distant lymph node metastasis, corresponding to M1a disease, was also more frequent in the CRPC N1 group than in the CRPC N0 group at CRPC diagnosis (76/213 [35.7%] vs 15/505 [3.0%], *P* < 0.001; Supplementary Table S1).

At Ra-223 initiation, the time from CRPC diagnosis to Ra-223 initiation did not differ between the two groups. The CRPC N1 group was younger and had higher PSA and ALP levels than the CRPC N0 group, whereas performance status ≥1, haemoglobin level, EOD score ≥ 3, and pain at Ra-223 initiation did not differ significantly between the groups.

### Descriptive supplementary analyses

Longitudinal changes in regional lymph node status and concomitant therapies during Ra-223 treatment are summarized in Supplementary Tables S2 and S3. These analyses were descriptive and showed no major imbalance in concomitant therapy patterns.

### Overall survival

OS from Ra-223 initiation was significantly shorter in patients with regional lymph node metastasis at CRPC diagnosis than in those without regional lymph node metastasis (Fig. 2). Median OS was 31 months (95% CI, 26–35) in the CRPC N0 group and 17 months (95% CI, 16–22) in the CRPC N1 group (log-rank *P* < 0.001).

**Figure 2:**
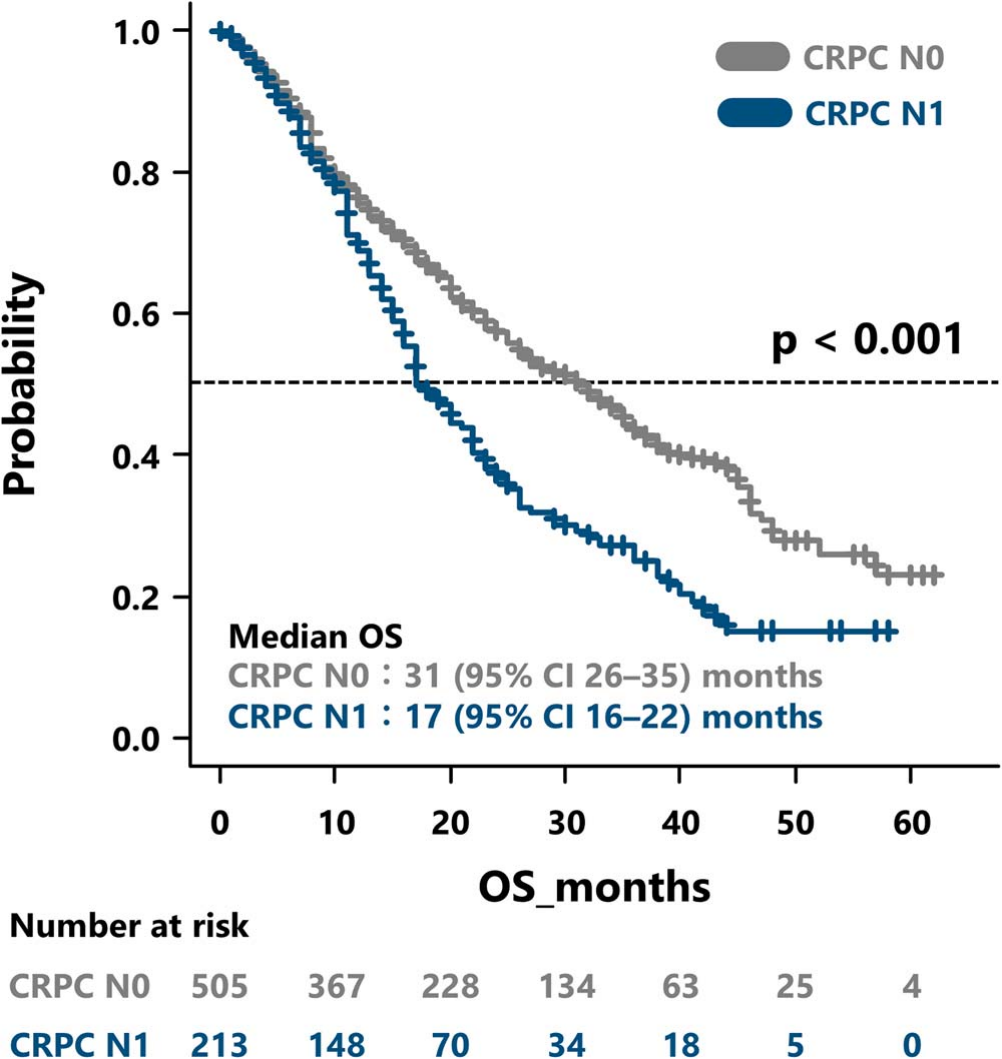
Overall survival from radium-223 initiation according to regional lymph node status at CRPC diagnosis. Kaplan–Meier curves for overall survival from Ra-223 initiation according to regional lymph node status at CRPC diagnosis. Overall survival was defined as the time from the first administration of Ra-223 to death from any cause or the date of last follow-up. Median overall survival was 31 months (95% CI, 26–35) in the CRPC N0 group and 17 months (95% CI, 16–22) in the CRPC N1 group. The univariable hazard ratio for regional lymph node metastasis at CRPC diagnosis was 1.595 (95% CI, 1.272–2.000). Survival curves were compared using the log-rank test. Abbreviations: CRPC, castration-resistant prostate cancer; OS, overall survival; Ra-223, radium-223.

### Cox regression analysis for overall survival

The results of the Cox proportional hazards regression analysis for OS are shown in Table 2. In the univariable analysis, regional lymph node metastasis at CRPC diagnosis was associated with poorer OS after Ra-223 initiation (hazard ratio [HR], 1.595; 95% CI, 1.272–2.000; *P* < 0.001). This association remained significant in the multivariable model adjusted for Gleason score sum, clinical T stage at diagnosis, performance status at Ra-223 initiation, EOD score at Ra-223 initiation, PSA at Ra-223 initiation, ALP at Ra-223 initiation, hemoglobin at Ra-223 initiation, treatment line of Ra-223, and age at Ra-223 initiation (HR, 1.396; 95% CI, 1.092–1.785; *P* = 0.008).

**Table 2:** Cox proportional hazards analysis for overall survival after radium-223 initiation.

Variable	Univariable HR (95% CI)	*P* value	Multivariable HR (95% CI)	*P* value
Primary exposure				
Regional lymph node metastasis at CRPC diagnosis, present vs absent	1.595 (1.272–2.000)	<0.001	1.396 (1.092–1.785)	0.008
Factors at initial diagnosis				
Gleason score sum, per 1-point increase	1.069 (0.945–1.208)	0.289	1.140 (1.002–1.297)	0.047
cT ≥3 vs <3	0.946 (0.739–1.210)	0.657	0.831 (0.640–1.078)	0.163
Factors at Ra-223 initiation				
Performance Status ≥1 vs 0	1.827 (1.463–2.282)	<0.001	1.514 (1.202–1.907)	<0.001
EOD score ≥ 3 vs <3	1.556 (1.216–1.992)	<0.001	1.012 (0.776–1.319)	0.931
PSA, per 10-fold increase	1.863 (1.652–2.101)	<0.001	1.589 (1.389–1.817)	<0.001
ALP, per 100 IU/L increase	1.205 (1.152–1.261)	<0.001	1.103 (1.042–1.168)	<0.001
Hemoglobin, per 1 g/dl increase	0.807 (0.745–0.875)	<0.001	0.909 (0.833–0.992)	0.033
Ra-223 as ≥3rd-line vs ≤2nd-line CRPC treatment	1.752 (1.408–2.179)	<0.001	1.314 (1.044–1.654)	0.020
Age, per 1-year increase	1.010 (0.995–1.025)	0.183	1.007 (0.992–1.022)	0.361

Hazard ratios were calculated using Cox proportional hazards regression models. Overall survival was defined as the time from the first administration of Ra-223 to death from any cause or the date of last follow-up. The multivariable model included regional lymph node metastasis at CRPC diagnosis, Gleason score sum, clinical T stage at diagnosis, performance status at Ra-223 initiation, EOD score at Ra-223 initiation, PSA at Ra-223 initiation, ALP at Ra-223 initiation, hemoglobin at Ra-223 initiation, treatment line of Ra-223, and age at Ra-223 initiation. Gleason score sum was modeled per 1-point increase. Clinical T stage was categorized as clinical T stage ≥3 versus <3 at diagnosis. Performance status was categorized as ≥1 versus 0. EOD score was categorized as ≥3 versus <3. PSA at Ra-223 initiation was log10-transformed; therefore, the hazard ratio represents the effect per 10-fold increase. ALP was modeled per 100 IU/L increase. Hemoglobin was modeled per 1 g/dl increase. Treatment line was categorized as Ra-223 administered as ≥3rd-line versus ≤2nd-line CRPC treatment. Age was modeled per 1-year increase.

The multivariable Cox model included 617 complete cases. Missing values in model covariates were as follows: Gleason score sum, *n* = 28; clinical T stage at diagnosis, *n* = 40; performance status at Ra-223 initiation, *n* = 34; PSA at Ra-223 initiation, *n* = 8; ALP at Ra-223 initiation, *n* = 10; haemoglobin at Ra-223 initiation, *n* = 12; and treatment line of Ra-223, *n* = 1. Some patients had missing values in more than one covariate.

Abbreviations: ALP, alkaline phosphatase; CI, confidence interval; CRPC, castration-resistant prostate cancer; EOD, extent of disease; Hb, hemoglobin; HR, hazard ratio; OS, overall survival; PSA, prostate-specific antigen; Ra-223, radium-223.

The multivariable Cox model included 617 complete cases. Missingness by covariate is summarized in the footnote to Table 2.

Given the imbalance in distant lymph node metastasis between the groups, we performed a sensitivity analysis by additionally adjusting the multivariable Cox model for distant lymph node metastasis at CRPC diagnosis, corresponding to M1a disease. The association between regional lymph node metastasis at CRPC diagnosis and poorer OS remained significant after this additional adjustment (HR, 1.341; 95% CI, 1.028–1.749; *P* = 0.030; Supplementary Table S4).

### Treatment outcomes and safety

Treatment course, response assessments, and safety outcomes according to regional lymph node status at CRPC diagnosis are shown in Table 3. Completion of six cycles of Ra-223 was less frequent in the CRPC N1 group than in the CRPC N0 group (141/213 [66.2%] vs 372/505 [73.7%], *P* = 0.043). Objective response on imaging was also less frequent in the CRPC N1 group than in the CRPC N0 group (17/170 [10.0%] vs 83/418 [19.9%], *P* = 0.004). PSA response, ALP decline ≥30%, disease control on imaging, pain improvement after Ra-223, symptomatic skeletal events, and grade ≥ 3 hematologic toxicities did not differ significantly between the groups.

**Table 3:** Treatment course, response assessments, and safety outcomes after radium-223 according to regional lymph node status at CRPC diagnosis.

Outcome	CRPC N0 (*n* = 505)	CRPC N1 (*n* = 213)	*P* value
Completed six cycles of Ra-223	372/505 (73.7)	141/213 (66.2)	0.043
PSA response	200/475 (42.1)	70/200 (35.0)	0.085
ALP decline ≥30%	217/505 (43.0)	102/213 (47.9)	0.226
Objective response on imaging (CR/PR)	83/418 (19.9)	17/170 (10.0)	0.004
Disease control on imaging (CR/PR/SD)	244/418 (58.4)	87/170 (51.2)	0.111
Pain improvement after Ra-223	19/446 (4.3)	9/188 (4.8)	0.768
Symptomatic skeletal event	71/489 (14.5)	29/206 (14.1)	0.880
Grade ≥ 3 neutropenia	30/461 (6.5)	10/179 (5.6)	0.666
Grade ≥ 3 anaemia	28/492 (5.7)	15/196 (7.7)	0.337
Grade ≥ 3 thrombocytopenia	8/492 (1.6)	2/196 (1.0)	0.733

Values are shown as n/N (%), unless otherwise indicated. Completion of Ra-223 was defined as completion of six cycles. PSA response was defined as a decrease of ≥50% from the PSA level at Ra-223 initiation. ALP response was defined as an ALP decline of ≥30% after Ra-223 initiation. Objective response on imaging was defined as complete response or partial response, and disease control on imaging was defined as complete response, partial response, or stable disease. Symptomatic skeletal event was defined as external-beam radiotherapy for skeletal symptoms, new symptomatic pathological bone fracture, spinal cord compression, or orthopedic surgical intervention for bone metastasis. Adverse events were graded according to the Common Terminology Criteria for Adverse Events version 5.0. Categorical variables were compared using the chi-square test or Fisher’s exact test, as appropriate.

Abbreviations: ALP, alkaline phosphatase; CR, complete response; CRPC, castration-resistant prostate cancer; CTCAE, Common Terminology Criteria for Adverse Events; PR, partial response; PSA, prostate-specific antigen; Ra-223, radium-223; SD, stable disease; SSE, symptomatic skeletal event.

Among patients who did not complete six cycles of Ra-223, reasons for discontinuation were available in 185 of 205 patients (90.2%) and are shown in Supplementary Table S5. Progressive disease was the most common reason for discontinuation in both groups.

### Cancer-specific survival

CSS from Ra-223 initiation was shorter in patients with regional lymph node metastasis at CRPC diagnosis than in those without regional lymph node metastasis (Supplementary Fig. S1). Median CSS was 35 months (95% CI, 29–39) in the CRPC N0 group and 20 months (95% CI, 17–23) in the CRPC N1 group (log-rank *P* < 0.001).

## Discussion

In this multicenter retrospective cohort study using the overall Ra-223-treated cohort of the FRONTIER study, regional lymph node metastasis at CRPC diagnosis was associated with poorer OS after Ra-223 initiation. This association remained significant after adjustment for Gleason score sum, clinical T stage at diagnosis, performance status, EOD score, PSA, ALP, hemoglobin, treatment line of Ra-223, and age at Ra-223 initiation. Patients with regional lymph node metastasis at CRPC diagnosis also had a lower completion rate of six cycles of Ra-223 and a lower objective response rate on imaging. These findings suggest that regional lymph node status at CRPC diagnosis may provide prognostic information before later Ra-223 selection.

Previous studies have identified several prognostic factors in patients with mCRPC treated with Ra-223. The ALSYMPCA trial established Ra-223 as a treatment that improves OS and delays symptomatic skeletal events in patients with mCRPC and bone metastases [1, 2]. Subsequent clinical and real-world studies reported that performance status, bone disease burden, ALP, hemoglobin, lactate dehydrogenase, PSA, pain, treatment line, and completion of Ra-223 were associated with survival outcomes [4–7, 10]. Some studies also included lymphadenopathy or lymph node metastasis near Ra-223 initiation or study registration as a prognostic variable [8, 9]. The present study differs from these reports by focusing on regional lymph node metastasis at CRPC diagnosis, which is an earlier point in the treatment course.

The timing of regional nodal assessment may be clinically relevant. Ra-223 is often selected after one or more systemic therapies for mCRPC, and disease assessment at Ra-223 initiation may reflect an already evolved disease state. In contrast, CRPC diagnosis is the point at which clinicians begin to plan sequential treatment for the castration-resistant phase. In the present study, patients with regional lymph node metastasis at CRPC diagnosis had more advanced disease features and a shorter interval from metastatic hormone-sensitive prostate cancer to CRPC. These findings support the interpretation that regional lymph node metastasis at CRPC diagnosis reflects a more aggressive disease phenotype rather than a treatment-specific determinant of Ra-223 efficacy.

The present findings should not be interpreted as evidence that patients with regional lymph node metastasis derive limited benefit from Ra-223. In ALSYMPCA, patients with malignant lymphadenopathy were eligible if the short-axis diameter was 3 cm or less, whereas patients with visceral metastases were excluded [1]. Therefore, nodal disease was not an absolute exclusion from the pivotal Ra-223 evidence base. In the present study, regional lymph node metastasis at CRPC diagnosis should be viewed primarily as a marker of higher-risk disease biology and poorer prognosis. It should not be considered a contraindication to Ra-223 or a direct determinant of treatment selection.

The distinction between regional lymph node metastasis and distant lymph node metastasis is also important. The primary exposure in this study was regional lymph node metastasis, corresponding to N1 disease at CRPC diagnosis. Distant lymph node metastasis, corresponding to M1a disease, was not included in the primary exposure definition. However, distant lymph node metastasis at CRPC diagnosis was more frequent in the CRPC N1 group than in the CRPC N0 group. This imbalance suggests that concurrent M1a disease and broader systemic tumor burden may have contributed to the poorer survival observed in the CRPC N1 group. In the sensitivity analysis additionally adjusted for distant lymph node metastasis at CRPC diagnosis, the association remained significant, although the effect estimate was slightly attenuated. These findings suggest that the main result was not explained solely by M1a imbalance; however, residual confounding by distant nodal disease and other markers of systemic tumor burden cannot be fully excluded.

The treatment landscape for mCRPC has expanded, and this context affects how regional nodal status should be interpreted. Prostate-specific membrane antigen (PSMA)-targeted radioligand therapy has become an important option for selected patients with PSMA-positive mCRPC after prior systemic therapy [11]. In addition, the PEACE-3 trial has supported clinical interest in combining Ra-223 with enzalutamide in patients with bone-metastatic mCRPC [3]. These developments suggest that patients with regional lymph node metastasis at CRPC diagnosis may require treatment planning within a broader systemic treatment sequence rather than assessment of Ra-223 in isolation. However, the present study did not directly evaluate the efficacy of concomitant enzalutamide, PSMA-targeted radioligand therapy, or any specific treatment sequence in patients with regional lymph node metastasis. Treatment selection should therefore not be based on regional nodal status alone.

The additional analyses provide context for the main finding but do not change its interpretation. Longitudinal assessment showed that regional lymph node status changed between initial diagnosis and CRPC diagnosis in a subset of patients, indicating that regional nodal disease can evolve during the disease course. Concomitant therapies during Ra-223 treatment, including androgen deprivation therapy (ADT) alone, enzalutamide, other androgen receptor signaling inhibitors (ARSIs), conventional hormonal therapy, chemotherapy, and bone-modifying agents (BMAs), were also summarized by regional nodal status. These results help describe the treatment context of the cohort. However, they should be interpreted descriptively because the present study was not designed to test whether a specific concomitant therapy modified the association between regional lymph node metastasis and survival.

This study has several limitations. First, the retrospective observational design may have introduced treatment selection bias and residual confounding. Patients with regional lymph node metastasis at CRPC diagnosis had more advanced disease features, and the multivariable model could not fully account for all differences in tumour biology, imaging findings, prior therapies, subsequent therapies, and systemic tumour burden. Second, distant lymph node metastasis was more frequent in the CRPC N1 group, and residual confounding by M1a disease may have affected the observed association with OS. Third, regional lymph node status at CRPC diagnosis was based on local investigator assessment. The specific imaging modality used to determine regional lymph node status was not captured in the study database, imaging modality and size criteria were not centrally standardized, and central radiologic review was not performed. These factors may have caused misclassification of regional nodal status.

Additional limitations should also be considered. The primary multivariable analysis used complete cases, and missing data may have affected the estimates if missingness was related to disease severity or survival outcomes. Nodal status at Ra-223 initiation was not systematically captured in the study database. Reasons for incomplete Ra-223 treatment were available in most patients who did not complete six cycles of Ra-223, but this analysis was descriptive because the reasons were collected retrospectively and were unavailable in 20 patients. CSS was a supportive endpoint, and cause-of-death classification may have varied across institutions. These limitations indicate that the findings should be validated in an external cohort or a prospective registry with standardized imaging assessment and detailed treatment-sequence data.

In conclusion, regional lymph node metastasis at CRPC diagnosis was associated with poorer OS after Ra-223 initiation in patients with bone-metastatic CRPC. This finding may provide prognostic information before later Ra-223 selection and may help inform treatment planning in the mCRPC course. Regional nodal status should be interpreted together with established prognostic factors, overall disease burden, M1a status, and treatment history. It should not be used alone to determine treatment selection.

## Supplementary Material

Figure_S1_hyag125

Main_document_JJCO_revise_20260705_table_s_hyag125

Supplementary_Figure_S1_caption_hyag125

## Data Availability

The data that support the findings of this study are available from the corresponding author upon reasonable request, subject to institutional and ethical restrictions.

## Permission to Reproduce Material From Other Sources

No material from other sources was reproduced in this manuscript.

## Clinical Trial Registration

Not applicable because this was a retrospective observational study.

## Institution Where the Work Was Performed

This study was performed as part of the Following Ra-223 in Outcomes after New-generation Therapy with Investigation of Effects in Refractory prostate cancer (FRONTIER) study, a multicenter retrospective cohort study conducted at participating institutions in Japan.
