# Supplementary material for: Prognostic impact of regional lymph node metastasis at castration-resistant prostate cancer diagnosis on survival after radium-223 treatment
Source: Jpn J Clin Oncol. 2026 Aug 7;56(11):1428–36. doi: 10.1093/jjco/hyag125 (PMC13766686; doi:10.1093/jjco/hyag125)
Supplement: Supplementary file 1 [file main_document_jjco_revise_20260705_table_s_hyag125.docx]

**Supplementary Table S1.**

**Additional patient characteristics and previous treatments according to regional lymph node status at CRPC diagnosis**

|  | CRPC N0 (n = 505) | CRPC N1 (n = 213) | p value |
| --- | --- | --- | --- |
| At initial diagnosis |  |  |  |
| Gleason score | 9.00 [8.00–9.00] | 9.00 [8.00–9.00] | <0.001 |
| N1, n/N (%) | 149/498 (29.9) | 181/212 (85.4) | <0.001 |
| Bone metastasis, n/N (%) | 297/505 (58.8) | 164/213 (77.0) | <0.001 |
| Visceral metastasis, n/N (%) | 32/505 (6.3) | 17/213 (8.0) | 0.425 |
| Distant lymph node metastasis, n/N (%) | 55/505 (10.9) | 75/213 (35.2) | <0.001 |
| At CRPC diagnosis |  |  |  |
| LATITUDE high-risk, n/N (%) | 221/354 (62.4) | 126/185 (68.1) | 0.191 |
| Distant lymph node metastasis, n/N (%) | 15/505 (3.0) | 76/213 (35.7) | <0.001 |
| At Ra-223 initiation |  |  |  |
| Previous abiraterone before Ra-223, n/N (%) | 178/505 (35.2) | 74/213 (34.7) | 0.897 |
| Previous enzalutamide before Ra-223, n/N (%) | 277/505 (54.9) | 128/213 (60.1) | 0.196 |
| Previous docetaxel before Ra-223, n/N (%) | 145/505 (28.7) | 84/213 (39.4) | 0.005 |
| Previous cabazitaxel before Ra-223, n/N (%) | 66/505 (13.1) | 39/213 (18.3) | 0.069 |
| Use of bone-modifying agents, n/N (%) | 346/495 (69.9) | 142/206 (68.9) | 0.800 |
| LDH, IU/L | 197 [169–234] | 194 [169–239] | 0.825 |
| CRP, mg/dL | 0.12 [0.05–0.39] | 0.19 [0.05–0.70] | 0.021 |

Values are shown as median [interquartile range] or n/N (%), unless otherwise indicated. Patients were classified according to the presence or absence of regional lymph node metastasis at CRPC diagnosis. Distant lymph node metastasis, corresponding to M1a disease, was not included in the primary exposure definition and was summarized separately as metastatic disease. Continuous variables were compared using the Mann-Whitney U test. Categorical variables were compared using the chi-square test or Fisher’s exact test, as appropriate.

Abbreviations: CRP, C-reactive protein; CRPC, castration-resistant prostate cancer; IQR, interquartile range; LDH, lactate dehydrogenase; Ra-223, radium-223.

**Supplementary Table S2.**

**Longitudinal changes in regional lymph node status from initial diagnosis to CRPC diagnosis**

| Initial diagnosis regional lymph node status | CRPC diagnosis regional lymph node status | n |
| --- | --- | --- |
| N0 | N0 | 349 |
| N0 | N1 | 31 |
| N1 | N0 | 149 |
| N1 | N1 | 181 |
| Unavailable | Not applicable for transition analysis | 8 |

Values are shown as n. This table summarizes transitions in regional lymph node status from initial diagnosis to CRPC diagnosis among patients with evaluable regional lymph node status at both time points. Patients were categorized as N0 to N0, N0 to N1, N1 to N0, or N1 to N1. Regional lymph node status at initial diagnosis was unavailable in 8 patients in the analytic cohort.

Abbreviations: CRPC, castration-resistant prostate cancer.

**Supplementary Table S3.**

**Concomitant therapies during radium-223 treatment according to regional lymph node status at CRPC diagnosis**

| Concomitant therapy during Ra-223 treatment | CRPC N0 group | CRPC N1 group |
| --- | --- | --- |
| ADT alone | 278/504 (55.2%) | 117/211 (55.5%) |
| Enzalutamide | 124/504 (24.6%) | 49/211 (23.2%) |
| Other androgen receptor signaling inhibitors | 12/504 (2.4%) | 2/211 (0.9%) |
| Conventional hormonal therapy | 87/504 (17.3%) | 35/211 (16.6%) |
| Chemotherapy | 1/504 (0.2%) | 3/211 (1.4%) |
| Bone-modifying agents | 346/495 (69.9%) | 142/206 (68.9%) |

Values are shown as n/N (%), unless otherwise indicated. Patients were classified according to the presence or absence of regional lymph node metastasis at CRPC diagnosis. Concomitant therapies during Ra-223 treatment included ADT alone, enzalutamide, other androgen receptor signaling inhibitors, conventional hormonal therapy, chemotherapy, and bone-modifying agents. Conventional hormonal therapy included estrogen therapy, estramustine, bicalutamide, and other non-enzalutamide and non-androgen receptor signaling inhibitor hormonal agents used during Ra-223 treatment.

Abbreviations: ADT, androgen deprivation therapy; ARSI, androgen receptor signaling inhibitor; CRPC, castration-resistant prostate cancer; Ra-223, radium-223.

**Supplementary Table S4.**

**Sensitivity Cox proportional hazards analysis additionally adjusted for distant lymph node metastasis at CRPC diagnosis**

| Variable | Hazard ratio | 95% confidence interval | p value |
| --- | --- | --- | --- |
| Regional lymph node metastasis at CRPC diagnosis | 1.341 | 1.028–1.749 | 0.030 |
| Distant lymph node metastasis at CRPC diagnosis | 1.152 | 0.820–1.618 | 0.416 |

The sensitivity model included the same covariates as the primary multivariable Cox model, with additional adjustment for distant lymph node metastasis at CRPC diagnosis, corresponding to M1a disease. The model included 617 complete cases. Hazard ratios were calculated using Cox proportional hazards regression.

Abbreviations: CI, confidence interval; CRPC, castration-resistant prostate cancer; OS, overall survival.

**Supplementary Table S5.**

**Reasons for discontinuation among patients who did not complete six cycles of radium-223**

| Reason for discontinuation | CRPC N0 (n = 125) | CRPC N1 (n = 60) |
| --- | --- | --- |
| Progressive disease | 69 (55.2) | 38 (63.3) |
| Adverse events | 27 (21.6) | 11 (18.3) |
| Patient request | 11 (8.8) | 4 (6.7) |
| Other | 15 (12.0) | 7 (11.7) |
| Unknown | 3 (2.4) | 0 (0.0) |

Values are shown as n (%). Percentages were calculated among patients with available reasons for discontinuation. Reasons for discontinuation were unavailable in 8 of 133 patients in the CRPC N0 group and 12 of 72 patients in the CRPC N1 group who did not complete six cycles of radium-223. “Unknown” indicates that the reason for discontinuation was recorded as unknown among patients with available discontinuation-reason data. This analysis was descriptive because reasons for discontinuation were collected retrospectively.

Abbreviations: CRPC, castration-resistant prostate cancer.
