# Supplementary material for: Prognostic impact of regional lymph node metastasis at castration-resistant prostate cancer diagnosis on survival after radium-223 treatment
Source: Jpn J Clin Oncol. 2026 Aug 7;56(11):1428–36. doi: 10.1093/jjco/hyag125 (PMC13766686; doi:10.1093/jjco/hyag125)
Supplement: Supplementary file 2 [file supplementary_figure_s1_caption_hyag125.docx]

**Supplementary Figure S1.**

**Cancer-specific survival from radium-223 initiation according to regional lymph node status at CRPC diagnosis**

Kaplan-Meier curves for cancer-specific survival from Ra-223 initiation according to regional lymph node status at CRPC diagnosis. Cancer-specific survival was defined as the time from the first administration of Ra-223 to death from prostate cancer or the date of last follow-up. Median cancer-specific survival was 35 months (95% confidence interval, 29–39) in the CRPC N0 group and 20 months (95% confidence interval, 17–23) in the CRPC N1 group. The univariable hazard ratio for regional lymph node metastasis at CRPC diagnosis was 1.623 (95% confidence interval, 1.298–2.029). Survival curves were compared using the log-rank test.

Alt text: Kaplan-Meier curve showing shorter cancer-specific survival after Ra-223 initiation in patients with regional lymph node metastasis at CRPC diagnosis than in those without regional lymph node metastasis.

Abbreviations: CI, confidence interval; CRPC, castration-resistant prostate cancer; CSS, cancer-specific survival; Ra-223, radium-223.
